# Parent-Implemented Telepractice Autism Intervention: A Case Study of Maintenance and Generalization

**DOI:** 10.3390/ijerph20031685

**Published:** 2023-01-17

**Authors:** Hedda Meadan, Michelle M. Sands, Moon Y. Chung

**Affiliations:** 1 Department of Special Education, University of Illinois, Champaign, IL 61820, USA; 2 Department of Special & Early Childhood Education, University of Wisconsin-Oshkosh, Oshkosh, WI 54901, USA; 3 Department of Education Studies, Stonehill College, North Easton, MA 02357, USA

**Keywords:** generalization, maintenance, parent-implemented intervention, telepractice

## Abstract

The extent to which people maintain new skills and generalize those skills to new contexts without support are two aspects of intervention research that can be difficult to examine, especially over a sustained period of time and across a variety of contexts. In past research, we have explored teaching parents and caregivers to implement evidence-based communication strategies with their young children with autism who are minimally verbal. When a former research participant contacted us with a request to participate in our project again, four years later and with a different son, we used this as an opportunity to ask questions about her maintenance of the skills in using the targeted strategies, and her generalization of those skills to a different child. Using the data collected with her older son, Ali, and new data collected four years later with her younger son, Rami, we present a case study of this mother. We discuss the implications of the findings on interpreting the efficacy of the telepractice intervention’s programming for generalization, identifying opportunities for refining the intervention, and insights useful for other intervention research.

## 1. Introduction

Parent-implemented autism interventions seek to increase parents’ and caregivers’ knowledge and skills for using evidence-based strategies with their children, with the ultimate goal of promoting the children’s development. Parent-implemented interventions are evidence-based practice [1], and parent and caregiver involvement in their children’s intervention is required by law [2] and recommended by organizations such as the Division for Early Childhood of the Council for Exceptional Children [3] and the National Association for the Education of Young Children [4]. Researchers have reported that parents can learn new strategies and skills and implement them with fidelity with their children in natural environments [5,6,7,8,9]. In addition, benefits for both parents and children have resulted from parent-implemented interventions conducted in person and via telepractice [10,11,12,13,14,15].

Many researchers have investigated the effectiveness of parent-implemented autism intervention using single-case research [16,17,18], a valuable and frequently-used methodology in special education. Its value, in part, is that it allows for experiments with a single or small number of participants, making it particularly useful in a discipline characterized by heterogeneity and individualization. In addition, single-case researchers have long emphasized that the extent to which changes maintain over time and generalize to new contexts are critical features of applied research [19,20,21,22]. In fact, in assessments of quality and outcomes of single-case research, consumers should consider generalization as a major component [23,24]. Generalization occurs when a behavior or skill learned in one context is used in a different context (e.g., across people, settings, stimuli, behaviors, or time). There are three main types of generalized behavior change: (a) setting generalization (e.g., different types of contexts), (b) response generalization (e.g., different types of responses), and (c) response maintenance (e.g., the behavior continues to occur after the teaching is ended, generalization across time) [25]. Stokes and Baer [26] emphasized that generalization should be programmed and not just expected. They proposed “an implicit technology of generalization”, including specific strategies that researchers and educators can use to promote generalization (p. 349). Yet, investigation on generalization and maintenance in autism research is limited [27,28]. Specifically, single-case research examples of parent-implemented intervention include limited maintenance data, e.g., [29,30,31], generalization data, e.g., [32,33,34], or both, e.g., [35]. When generalization and maintenance data are reported, the types of generalization and/or maintenance vary widely. In their literature review of 11 studies of parent-implemented communication interventions, Lang and colleagues [12] found only six studies that reported generalization data and seven that reported maintenance data, and these data measured various aspects of generalization and maintenance. Similarly, Gerow and colleagues [36] reviewed 26 studies on parent-implemented functional communication training and found that only 15 studies included generalization and maintenance data on caregivers’ skills, which also varied widely.

Pennington and colleagues [37] reported challenges in comparing generalization measured across studies. One barrier is the use of different terms and definitions for generalization and maintenance by different researchers (e.g., maintenance, follow-up, or fading). Another challenge is the variation in measurements. Numerous studies have measured generalization across settings, typically examining if the target behavior generalized from a clinic or school to home, e.g., [30,33,38,39,40,41,42,43,44]. However, few studies have measured the extent to which a parent or caregiver generalized the targeted skill to other children when two or more children could benefit from the same intervention. Boyle et al. [45] measured the generalization of a caregiver’s use of an intervention to address the challenging behavior of a sibling of the child who participated in the intervention. They found that, in one of two settings (i.e., bedtime), the parent generalized the use of parenting strategies from one sibling to another, and that it resulted in lower levels of disruptive behavior from both children. Subramaniam et al. [44] measured the generalization of a caregiver’s implementation of discrete trial instruction from practice with an adult to applied use with their child. The caregivers were provided the opportunity to implement discrete trial instruction with an adult in the baseline phase and low implementation fidelity was observed. Following in vivo training, implementation fidelity in the adult was 90%. The researchers then conducted a generalization probe, in which the caregivers implemented discrete trial instruction with their child, and all four parents met the 80% mastery criteria.

Generalization has been measured in various phases (baseline, intervention, post-intervention, and follow-up) and for differing numbers of probes. Stokes and Baer [26] argued that generalization should be programmed and measured, and Pennington et al. [37] stated that the “decision about measurement [of generalization and maintenance] should be purposeful and guided by data” (p. 21). There are no specific guidelines for the number of generalization probes a researcher should collect during the study (e.g., generalization across contexts) and after the intervention is completed (i.e., maintenance). However, when developing an intervention, researchers need to identify to which contexts the behavior should be generalized (e.g., different settings, different children) and measure the behaviors in these contexts. At a minimum, generalization probes should be collected during the baseline, intervention, and follow-up phases. Without having a generalization probe in the different study phases, it is difficult, if not impossible, to evaluate changes in the different contexts as they relate to the intervention.

To establish that a caregiver has learned the target skill and maintained its use (i.e., generalization across time), a sufficient number of maintenance probes must be collected over time. Many parent-implemented interventions collect only two or three maintenance probes during the follow-up periods that occur within six months of the end of the intervention [12,36]. Some researchers have measured the maintenance of parent-implemented interventions beyond six months post-intervention. For example, in Binnendyk and Lucyshyn’s [46] study on the effectiveness of a family-centered positive behavior support approach to reduce food refusal behavior in a child diagnosed with autism, improvements were reportedly maintained for up to 26 months post-intervention. The maintenance data were collected one week, five weeks, six weeks, and 26 months after the intervention ended. However, at the 26-month follow-up, not all of the parent-child feeding routines were successfully completed. Because long-term maintenance data are so rarely collected, researchers’ understanding continues to be limited as to whether intervention effects that need to be maintained are, in fact, maintained over long periods of time when sustained maintenance is likely to be beneficial.

There is a benefit to evaluating the generalization and maintenance of outcomes in parent-implemented interventions. In fact, Kennedy [21] suggested that maintenance is one measure of the social validity of the intervention; if the learned skills and strategies are not generalized appropriately and when needed to different settings, children, and beyond, and are not maintained over time, it is possible that the goals, procedures, and outcomes of the intervention were not socially important.

Given the importance of considering maintenance and generalization and the challenges inherent to exploring them, we present a case study comprising data collected across two time points within the same family. Specifically, we asked: (1) In what ways and to what extent did one mother maintain and generalize the skills learned during training and coaching as part of a parent-implemented autism intervention? (2) How do findings about her maintenance and generalization inform our understanding of the intervention’s effects?

## 2. Case Report

### 2.1. Participants

Mediha was a married woman in her 30s and mother to three sons. She and her husband immigrated to the U.S. from the Middle East and lived in a small city near a university. Mediha had a bachelor’s degree, worked full-time as a mother and homemaker, and volunteered routinely in the community. Rami, Mediha and her husband’s third and youngest son, was aged 4; 11 (years; months) at the start of the case study. Rami had been diagnosed with autism and attended the local public preschool five days per week and received additional speech-language therapy (1 hr per week) and occupational therapy (30 min per week) at their local hospital. At the beginning of the study, Mediha reported that Rami understood and said 22 words (13th percentile) on the MacArthur-Bates Communicative Development Inventories (CDI) [47]; at the end of the study (i.e., six months later), she reported that he understood and said 72 words (40th percentile). At the beginning of the study, Mediha reported that he relied primarily on behaviors, gestures, and imitating spoken models to communicate. For example, she explained in her pre-intervention interview,

*“if he wants something, then he will grab my hands and then move me to where he wants help with or what he wants out of the fridge, …grabbing my hands and taking me to the fridge… Then, I sort of acting like eat, [and say] ‘do you want eat?’ And then he will repeat the word ‘eat’ after me.”*

Four years after the completion of a parent-implemented autism communication intervention [14] with her older son, Ali, Mediha reached out to the researchers after her youngest son, Rami, was diagnosed with autism and demonstrated similar characteristics as his brother Ali. She stated that the targeted communication strategies were helpful for Ali, but she wasn’t sure how to use the same strategies with Rami and felt she could benefit from additional support from the research team. The research team had had no contact with Mediha in the intervening time and, therefore, had no data about what support she received in those four years. Instead, building off the data collected four years earlier, we conducted a case study [48] of Mediha and her family, collecting behavioral observation, self-report, and interview data to inform our exploration of Mediha’s maintenance and generalization of skills she had learned during her participation in the original parent-implemented autism intervention study.

### 2.2. Design

The case study comprised data collected across two time points with the same family in which behavioral observation data, interview, and self-report data were collected. The program used (i.e., the independent variable) consisted of two sequential components: training, followed by coaching. The training was asynchronous, self-directed, self-paced online modules. Mediha was asked to complete a total of five modules sequentially (i.e., an overview of the program and four modules focused on each targeted strategy). Four evidence-based communication strategies were targeted: (a) environmental arrangement, (b) modeling, (c) the mand-model, and (d) time delay. Each module contained a 9- to 14-min video, a short check-for-understanding quiz, and a downloadable flowchart of the strategy steps. The modules were programmed to support generalization through the use of multiple examples of the strategies across settings, routines, items, and vocabulary.

Then, a researcher provided synchronous coaching via telepractice. The coaching session format was based on Rush and Shelden’s [49] framework and included: (a) pre-observation, developing an action plan and reviewing steps for implementing the targeted strategy; (b) observation, observing the parent-child interaction without interrupting; and (c) post-observation, asking Mediha to reflect on the session and the coach providing supportive and corrective feedback (see additional description of coaching procedures in Meadan et al. [14]). All the study activities were conducted in Mediha’s home during her regular routines and activities with Rami. The researchers did not visit her home in person but conducted all contact via telepractice.

Mediha completed all the training module components online (i.e., 100% fidelity) and her fidelity of implementation for coaching was measured by a 10-item checklist, which was completed by the coach during each coaching session. A doctoral student in special education, who was not involved in the study, served as an independent observer for checking the fidelity of implementation (i.e., reliability check). All the coaching sessions were conducted with 100% fidelity. We also calculated the reliability of the fidelity of implementation for 33% of the coaching sessions, and point-by-point agreement was calculated by counting agreements on the checklist, divided by the agreements plus disagreements multiplied by 100, for a reliability score of 100%.

### 2.3. Behavioral Observation Data

Behavior observation rating data were collected on Mediha’s implementation of each of three strategies (i.e., modeling, the mand-model, time delay). Each strategy was established as a four-step task analysis. For example, when Mediha used modeling, she needed to (a) establish joint attention, (b) provide a verbal model, (c) wait 2–3 s for the child to imitate her model, and (d) use the strategy again or provide verbal feedback based on how the child responded. Thus, the fidelity with which Mediha used each strategy in each trial was scored on a 4-point scale. A score of 4 indicated high-fidelity strategy use (i.e., all four steps in the strategy were implemented correctly); scores of 1, 2, or 3 indicated one or more steps were missed or implemented incorrectly (low-fidelity strategy use). For more information about the coding, see Meadan et al. [14]. Videos were recorded approximately once a week, although the schedule of video recordings varied based on Mediha’s schedule. The duration of each video varied, as the recordings were based on the length of time Rami was participating in the routine she chose (range 3–28 min). For each observation session, a 3-min portion of the video was randomly selected, using an online random number generator, and coded.

#### 2.3.1. Analysis

As the purpose of this case study was to explore generalization and maintenance rather than establish a functional relation, the data from the intervention with Ali [14] and the data from the intervention with Rami are presented in the graph. The rate of high-fidelity and low-fidelity strategy uses was graphed using a stacked bar graph and visually analyzed to identify patterns in the maintenance and generalization of Mediha’s strategy use with both of her sons.

#### 2.3.2. Interobserver Agreement (IOA)

The first and second authors observed and coded all the data. Before assessing the IOA, the observers, who had used the same coding procedures in other studies, reviewed the coding procedures and coded a small subset of the sessions, compared their results, and discussed disagreements; these sessions were excluded from the calculations. The categories coded included (a) the timestamp of the event on the video (i.e., Mediha’s strategy use or child communication), (b) the type of strategy (i.e., modeling, the mand-model, or time delay), and (c) the fidelity of the strategy use (score of 1–4). Agreements were defined as both observers coding each of the three categories in the same way; for the time of the event, a window of 3 s was permitted. The IOA was calculated as agreements divided by the agreements plus disagreements, multiplied by 100. One author coded all the sessions, and the second author independently coded at least 33% (range 33–40%) of the sessions, selected at random, in each phase (i.e., baseline, post-training, coaching, maintenance). The overall agreement across all the categories was 90.9%. Table 1 contains the IOA averages and ranges for each phase.

### 2.4. Self-Report and Interview Data

Mediha was asked to complete an intake questionnaire to report on the ways she supported Rami’s communication, their typical routines, and her goals and priorities for Rami’s communication development. Then, during the coaching, Mediha completed a self-report form before or at the beginning of each coaching session indicating (a) how often she used each strategy each day, (b) during what routines and activities she used them, and (c) her confidence in using the strategies on a five-point scale from “not confident” to “very confident”. In addition, the second and third authors interviewed Mediha at the beginning of the study (i.e., pre-intervention interview), and the second author interviewed her again after she completed the training, and again at the end of the study. The interview protocols are available in Table 2.

#### Analysis

The interview transcripts and self-report data were compiled and reviewed by the research team. The team identified themes across the data sources. Then, they reviewed the data sources and results from Mediha’s responses in the first study to explore similarities and differences between the two time points. This information was integrated into our analysis of the graphed behavioral observation data to inform our interpretation of how the intervention impacted Mediha’s generalization and maintenance of the strategy use.

## 3. Findings

**Figure 1:**
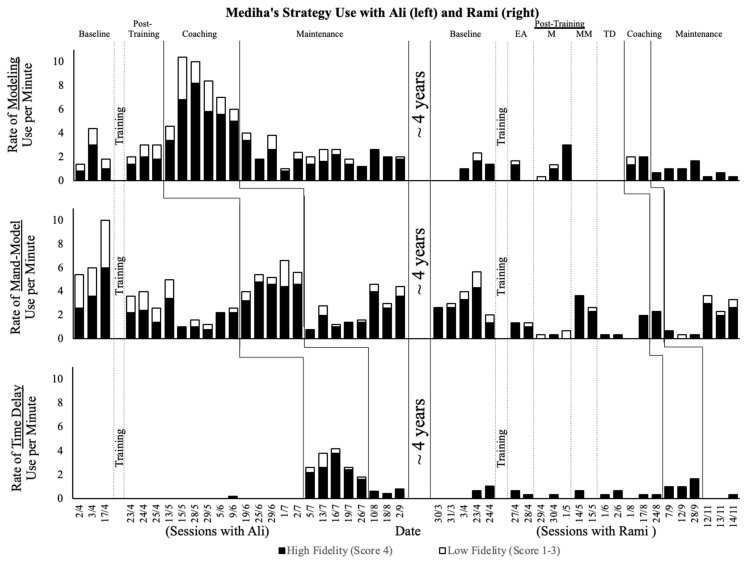
Mediha’s observed behavioral data with the approximate 4-year gap between studies marked with two vertical lines and labeled. Each tier of the graph represents one strategy, with modeling on the top tier, the mand-model on the middle tier, and time delay on the bottom tier. The height of the bars represents the rate per minute of strategy use during the session. Black portions of each bar represent high-fidelity strategy use and white portions represent low-fidelity strategy use.

In Figure 1, we present Mediha’s observed behavioral data from both time points in a single bar graph, with the approximate 4-year gap between the studies marked with two labeled vertical lines. Each tier of the graph represents one strategy, with modeling on the top, the mand-model in the middle, and time delay on the bottom tier. The height of the bars represents the rate per minute of strategy use during the sessions. The black portions of each bar represent high-fidelity strategy use, and the white portions represent low-fidelity use.

During this case study, when we collected the baseline data on Mediha’s use of the previously studied communication strategies with her youngest son, Rami, she used all three strategies with high fidelity in the baseline phase, an indication that she had maintained these skills over the past four years and had generalized the strategy use to her interactions with Rami. Although she used the strategies at much lower rates with Rami than when used with Ali, this was not a concern, because Mediha had been encouraged to follow her children’s lead and use the strategies in naturally-occurring routines. This meant that the variability in rate was expected and that high-fidelity use, regardless of how often a strategy was used, was the primary goal. Given the high proportion of high-fidelity strategy use in the baseline conditions, we could not conduct a second true single-case experiment with Mediha and Rami, as Mediha had already reached the performance criterion in the baseline (i.e., at 80% high-fidelity use). However, in the interview and on her self-reports, Mediha reported that she did not feel confident using these strategies with Rami and felt she needed a deeper understanding of the strategies to implement them effectively with him. During her pre-intervention interview, she said,

*“I learned a lot from what Ali went through, and, I guess, with what I’ve learned, I can maybe help Rami. I know that I still have much more to learn. … When [my coach] would tell me something before [first study], I would just try to do whatever she says and that’s it. But now [current study] …maybe I will be asking more questions about what [my coach] is telling me to do, [I will ask for] more details. And maybe, maybe it will help make it better. Maybe. Or maybe I will just gain a lot of knowledge for myself.”*

Thus, we gave Mediha access to the online training modules that presented the same content as the first study but delivered them asynchronously via online modules.

After completing each online training module on the targeted strategies, Mediha submitted a video(s) of her interactions with Rami. These videos are separated by dotted vertical lines in Figure 1 that indicate what portion of the training she had completed when she submitted the video. By visually analyzing these data, we observed variability in the rate with which Mediha used each strategy with Rami, but she continued to use all three strategies with similar, high proportions of high-fidelity use. In her post-training interview, however, Mediha expressed a continued lack of confidence in her strategy use with Rami. She reported that she was using the strategies “a lot at home, even away from the cameras and the videotaping [for the study]. I try to implement it as much as possible.” However, her first concern was that she was interacting unnaturally with Rami. She said,

*“I notice that sometimes when I play with Rami, …I feel like I am a robot because I’m focusing on the speech. … [I want] ideas about how to play, how to make it work, …how I can, if something fails, how I can fix it or what I need to kind of not focus on.”*

Her second concern was that she was not appropriately selecting vocabulary and/or the strategy best suited to support Rami in a particular situation or with particular vocabulary. During the post-training interview, she explained,

*“Sometimes I do feel like I am missing which word he’s supposed to say before which word. Like, would it be better if I would have him say, ‘More, please’ or ‘More bubbles’ or just ‘more’ or just ‘bubbles.’ Which one as a sequence would be better for a child to learn? … So, I’d say [that’s] the one that I would need more support with… and which strategy will work better in order to get this. This will be a big help for Rami and for me.”*

These concerns mark a distinct shift in her personal goals while participating with Rami from the goals she had with Ali. In both studies, she expressed that her motivation for participating was to support both Ali and Rami in developing strong verbal and social communication skills. During her intake interview in the first study, she indicated that her goal was to learn “more strategies to get [Ali] out of silence,” and she expanded on this in the current study, saying,

*“I remember what I told you before about Ali, that I just want Ali to be saying his basic needs. I’m not going to say that now about Rami. I’m actually looking for more …more as [in] comprehension, I would say, more about their academic writing and being able to express themselves. Not just his basic needs, but more than that.”*

Her expressions of confidence or lack of confidence were no longer connected to her previous goal of simply learning new strategies and implementing them correctly to help her children say words. In the current study, she was seeking to implement the strategies with sophistication to help Rami communicate expansively. This shift in her definition of what strategy use would accomplish altered the context under which she rated her confidence, and this aligned with the trends we were observing in her behavioral data; she had maintained and generalized high-fidelity implementation. Now, she was seeking to, more expertly, wield the strategies to support her son.

After she had completed the online training modules, we then provided her with coaching. Given that Mediha had maintained and generalized high-fidelity use of the strategies, the coach focused her feedback on building Mediha’s confidence in using the strategies with Rami in natural and child-directed ways, and in helping her develop a deeper understanding of how the strategies worked. The coaching procedures included: (a) a pre-observation conference during which the coach and Mediha discussed her practice with the strategies since the last coaching session, completed a confidence rating about using the strategies, reviewed the steps in one focus strategy (i.e., modeling, the mand-model, or time delay), and jointly developed a goal for the observation; (b) action and observation, in which Mediha used the focus strategy while she engaged with Rami in one of their regular routines (e.g., snack, playtime), while the coach silently observed via videoconference; and (c) a post-observation conference in which Mediha reflected on her use of the focus strategy and the coach provided both supportive and corrective feedback. At the beginning of each coaching session, the coach asked Mediha to rate her confidence with the strategy; these self-reported ratings served as a starting point for Mediha to explain to her coach areas of focus for providing feedback. Table 3 displays Mediha’s self-reported confidence ratings during coaching.

During the interactions with Rami, Mediha continued to use each strategy with high fidelity and at rates similar to those in the baseline phase and during training with Rami (see Figure 1). In addition, her self-reported confidence quickly went from ratings of “confident” or “fairly confident” during the first three coaching sessions (and one self-report on a day she did not receive coaching) to “very confident” across all the strategies (see Table 3). Her high-fidelity use of the strategies persisted during the maintenance phase (see Figure 1). In an interview conducted in the maintenance phase, Mediha explained what the coaching had contributed to her strategy use with Rami:

*“Well, what worked for me [is that my coach] really gave me some great time and hints. … I was like, I’m doing the same strategy over and over, but I don’t see the results right away. But when I would follow her guide and with those tips and hints, it kind of makes things way easier than the way it is. … I learned that time delay won’t come unless I use the three strategies and keep being very consistent with them, and then, after that, that the delay and response will come. … So, this is one thing that I literally, I was able to see that, when [coach] was training me for Ali. In the beginning, I was really, I would say, mad about how many times I have to do modeling and how many times I need to do mand-model. I was just like, ‘This is too much!’ But, actually, in the last session, she [coach] told me we’ll do mand-model, that we’ll do time delay after… and I saw my child, where he’s at, and he was really able to do that time delay thing. That was like, ‘She was right. She knew what she was supposed to do. She knew how many times I need to keep going with mand or that model so he would be able to come up and initiate those words while I’m waiting for him to respond. … After I got the second time [current study] coaching, I was very comfortable and maybe that’s why I would [use the strategies] as a more nature (sic) in my life.”*

## 4. Discussion

We had a unique opportunity to examine how a parent maintained and generalized communication teaching strategies over a four-year period and across two children who had both received an autism diagnosis. We found that Mediha maintained and generalized her skills, as measured by behavioral observation. She maintained her skills in high-fidelity strategy use over a four-year period and generalized the skills she had learned with her older son, Ali, to her younger son, Rami (see Figure 1). However, a closer look at other case study data (i.e., self-reports and interviews) exposed the complexity inherent to applying, maintaining, generalizing, and adjusting the skills. Mediha’s observable behavior (i.e., high-fidelity communication strategy use) was not what propelled her to seek the research team’s assistance with Rami. Instead, in the current study, she sought affirmation that she was using the strategies appropriately with Rami and additional guidance in more expertly and naturally wielding them.

While her actual skills in implementing the strategies with fidelity did maintain and generalize, what did not maintain was the “soft” skill of feeling that she was doing the right things to support her child. In addition, as is often the case after mastering a new skill, Mediha reported that she realized how much more she could learn about the nuanced use of communication teaching strategies once she had learned their basic implementation.

Perhaps the most important finding we see in our experience with Mediha is that having someone come and affirm and/or support her efforts to support her children’s communication, provide individualized and timely feedback, and provide general support were extremely important contributions to the program for Mediha. First, although Mediha’s observable and measurable skills in using the strategies with high fidelity maintained and generalized over time and across children (a positive and desired result for parent/caregiver-implemented intervention), the emotional support that she received from those initial services with Ali did not persist, and she still needed and sought affirmation and support in the current study with Rami, even with her strong technical skills. Second, the sophisticated use of any skill comes with ongoing feedback, reflection, and learning [49,50] Mastering basic skills with Ali led Mediha to want more information about how to use the strategies she had learned in more natural ways and with more nuance in the current study. In short, Mediha sought support for both of her autistic sons, in spite of the skills she had learned with Ali that she had successfully maintained over time and generalized to her parenting of Rami. Finally, naturalness in using the strategies was important to Mediha, and strategies based on applied behavior analysis are frequently (and, often, rightfully) criticized for, as Mediha put it, making parents feel like “robots” or constantly serving as their child’s therapist rather than parent, e.g., [51]. Perhaps, if the research team had more strongly emphasized the need to incorporate strategies such as following the child’s lead and scaffolding with Ali, her confidence and comfortability would have been improved for both children, and our focus in the current study could have shifted to different strategies and increased sophistication. Giving families room to define the nature and shape of their interactions with their children is of paramount importance.

## 5. Limitations

As with any research endeavor, this study has several limitations that should be considered. First, we do not have information about the other supports Mediha may have received in the four years between the studies that may have helped her maintain and generalize her skills. Future research should examine what conditions facilitate such long-term maintenance and generalization. It might also be important to collect additional information about parental and family outcomes (e.g., stress level, quality of life). Second, we based our assessment of the appropriateness of Mediha generalizing the strategies to her other son (Rami) on communication assessments alone. While we think this was sufficient for this intervention, not every skill should be generalized to every other situation, especially to other children, as they have unique and individual needs and preferences. Examinations of generalization should take into account whether generalization to a particular context or across an extended period of time is indeed appropriate. Finally, the methodology used, the case study, does not allow an examination of function relation. Future researchers might want to employ experimental design to better understand the generalization and maintenance of parent-implemented autism interventions.

## 6. Conclusions

In spite of the encouraging evidence that Mediha maintained and generalized high-fidelity strategy use, she still wanted and sought support with her youngest autistic son. Early intervention, home visiting (in person or via telepractice), and, indeed, all special education supports can be beneficial to parents/caregivers, regardless of their previous access to support or their existing skills. However, such support is likely to be useful only if practitioners attend to people’s opinions and their subjective perceptions of the value of the support provided (i.e., social validity; [52]). Services, after all, should bias someone’s opinion of the support they have received, for they now have an experience upon which to base their position. Decisions based solely on the data presented in Figure 1 would have led the research team to tell Mediha she was doing a great job with Rami and to keep doing what she was doing. Only through conversation and the family–professional partnership did we uncover why, with her maintained and generalized skill, she still sought to repeat the program. This case study points to the extent to which the program was effective in programming for maintenance and generalization, and the ways in which it was socially valid in Mediha’s estimation. Mediha’s story may be useful to researchers and practitioners seeking similar outcomes, as it may readily transfer to many other contexts in research and practice.

## Figures and Tables

**Table 1:** Interobserver Agreement by Phase.

	Average Percent of IOA by Coded Category (Range)		
Phase (*n*, % of Sessions Coded)	Timestamp	Strategy Used	Fidelity Score
Baseline (2, 40)	81.3 (79–83)	100	95.5 (92–100)
Post-Training (3, 33)	79.4 (67–100)	96.0 (90–100)	88.0 (71–100)
Coaching (2, 33)	90.5 (88–92)	100	100
Maintenance (1, 33)	83.3	100	100
**Overall**	**82.8**	**98.7**	**93.5**

**Table 2:** Interview Protocols.

**Pre-intervention Interview Protocol**
Tell me about you and your child (e.g., daily schedule, routine).
Tell me about (child’s) communication skills and behavior.
How does your child let you know his preferences? (e.g., what he likes/doesn’t like)
How does your child get your attention to communicate something he has noticed?
Tell me how you and other family members communicate with him.
How does your child communicate in different settings, such as the park or library (or wherever you spend time)?
What seems to help your child communicate? Gestures, signs, pictures, etc.?
Does [child] like to play with you or other adults/children?
What are some things [child] likes and dislikes?
Are there challenges regarding [child]’s communication skills/behavior?
What strategies do you use at home to promote/enhance [child’s] communication skills?
What are your wishes for your child?
What are your hopes and dreams for [child] relative to communication skills? What do you hope the program will do for [child]?
What do you hope the program will do for you and other members of your family?
In general, how do you advocate for your child?
**Post-intervention Interview Protocol**
Tell me about your experience so far with the project.
Can you tell me about the activities you have been doing with your child since you started the project?
Did you notice any changes in the way your child communicates with you and others in the family since you began?
Did you notice any changes in your family since the project began?
Describe your overall perspective on the activities you had to do for the project.
Describe your overall perspective on the goals of the project.
Please describe what was effective about the intervention (i.e., training and coaching). What were the best features of the project? Why?
Please describe what was ineffective about the intervention (i.e., training and coaching).
Is there anything you would change about the project?
Describe your relationship with [your coach].
Please describe your experience using technology for the project (e.g., Skype, shared iCal, forms, Box, iPad)
Describe your overall perspective on the outcomes of the intervention, for you, your child, and your family.
Did the project impact your experience with Early Intervention services?
Would you recommend that Early Intervention service providers use coaching? Why or why not?
Would you recommend the program strategies (modeling, mand-model, time delay, environmental arrangement) to other parents/caregivers? Why or why not?
If another service provider asked you to complete online training modules on different strategies that were similar in format to the ones you completed for the project, would you be interested? Why or why not?

**Table 3:** Mediha’s Self-reported Confidence Ratings During Coaching.

Phase	Strategy		
	Modeling	Mand-Model	Time Delay
Coaching on Modeling			
8/1	Confident		
8/17	Fairly confident		
Coaching on Mand-model			
8/24	Very confident	Confident	
9/1 *	Fairly confident	Confident	
Coaching on Time Delay			
9/7	Very confident	Very confident	Very confident
9/12	Very confident	Very confident	Very confident
9/14 *	Very confident	Very confident	Very confident
9/15 *	Very confident	Very confident	Very confident
9/28	Very confident	Very confident	Very confident
10/2 *	Very confident	Very confident	Very confident

Note. Self-reports from generalization probes on days when Mediha did not receive coaching are marked with an asterisk.

## Data Availability

Data are available upon request from the first author.
